# Clinical Repeatability and Inter-Method Agreement of VITA Easyshade Advance 4.0 and Rayplicker Cobra for Tooth Shade Determination: An Exploratory Cross-Sectional Study

**DOI:** 10.3390/diagnostics16172775

**Published:** 2026-08-29

**Authors:** L. Portero-Ruz, M. Valor-Priego, A. Martín-Vacas, M. M. Paz-Cortés, J. Mena-Álvarez, C. Rico-Romano

**Affiliations:** 1 Faculty of Dentistry, Alfonso X el Sabio University, 28691 Madrid, Spain; lporruz@myuax.com (L.P.-R.); mprieval@uax.es (M.V.-P.); amartvac@uax.es (A.M.-V.); cromaric@uax.es (C.R.-R.); 2 Faculty of Dentistry, Complutense University of Madrid, 28040 Madrid, Spain; mapaz08@ucm.es

**Keywords:** colour measurement, dental colour, Easyshade, human eye, Rayplicker Cobra, spectrophotometer

## Abstract

**Background:** Instrumental shade determination may reduce some sources of subjectivity associated with visual shade selection, but device repeatability and interchangeability remain clinically relevant concerns. **Objective:** The aim of this study was to evaluate the reproducibility and clinical utility of two spectrophotometric devices (Easyshade and Rayplicker Cobra) for tooth shade measurement and selection. **Methods:** In total, 35 adults were evaluated at seven predefined maxillary tooth regions: the cervical, middle, and incisal thirds of teeth 2.1 and 2.3, and the middle third of tooth 2.6. Each instrumental measurement was repeated three times, with complete removal and repositioning of the device between readings. Visual shade selection using the VITA Classical guide was performed once per site by the same trained operator, whose normal colour vision had been confirmed before data collection. Teeth were kept hydrated, patients remained in the same position, and both devices were calibrated according to the manufacturers’ instructions. Intra-device strict repeatability and inter-method agreement were assessed descriptively and using Cohen’s Kappa, as appropriate. **Results:** Descriptive pooled strict repeatability was 52.2% for Easyshade and 53.1% for Rayplicker Cobra. Repeatability varied by tooth and region. Inter-device agreement was predominantly slight to fair (0.053–0.386). Easyshade showed moderate-to-substantial agreement with the visual comparator in selected regions, with the highest Kappa in the middle third of tooth 2.1 (0.619), whereas Rayplicker Cobra did not exceed fair agreement (maximum 0.362). **Conclusions:** Both spectrophotometers showed similar descriptive strict repeatability percentages; however, their low inter-device agreement indicates that their categorical shade outputs should not be considered interchangeable. The greater agreement of Easyshade with VITA Classical in selected regions reflects inter-method comparability rather than superior trueness, as no independent instrumental reference standard was used.

## 1. Introduction

Colour in dentistry is especially important when performing dental restorations. Choosing the right colour, consistent with the surrounding teeth and the patient’s needs, allows us to achieve work that conveys harmony, balance and naturalness [1].

Colour perception is described along three dimensions: value, hue, and chroma [2,3], and depends on the interaction between a light source, the object, and the observer’s retinal photoreceptors [4]. These dimensions underlie the Munsell system and its modern descendants, including the CIELAB colour space [5]. Clinically relevant sources of variability include the colour temperature of the operatory light—dental units typically provide light around 4000 K, whereas 5500 K, close to natural daylight, is recommended for shade matching [6]—context- and illumination-dependent shifts in colour perception [7], the translucency, refraction, and layered optical behavior of enamel and dentine—with dentine being the principal determinant of tooth value and chroma [8,9,10,11,12]—and metamerism—whereby the same tooth can appear a different shade under different illuminants, a phenomenon also documented between natural teeth and ceramic restorations [9,13,14]. Environmental factors such as background colour, patient clothing, and cosmetics can further bias visual shade assessment and should be minimized or standardized during shade taking [10,11].

Colour depends on several factors, including light, object characteristics, and observer characteristics [15,16]. This makes colour a subjective quality that is difficult to determine in a standardized and reproducible manner. Subjective methods, such as dental guides, are still used in clinical practice, but objective methods have emerged with the idea of solving the problem of subjectivity. The background presented in this Introduction is narrative rather than systematic in nature; it focuses primarily on the instrumental (spectrophotometric) methods of shade determination, since these are the technologies directly evaluated in the present study, and addresses the visual method only insofar as it is relevant for comparison. These instruments measure the reflection of light obtained after the beam hits the tooth. There are colorimeters and spectrophotometers: colorimeters measure light absorption through the reflection of the emitted light collected by the instrument, and spectrophotometers measure the quality and quantity of colour through the reflection collected after the incidence of a defined emitted light. The Vita Easyshade Compact^®^ (VITA Zahnfabrik, Bad Säckingen, Germany) is a wireless digital spectrophotometer composed of three spectrophotometers: one spectrophotometer emits light, and the other two measure the reflections emitted by the object by comparing the wavelengths of the dispersions produced (refer to the shades of the Vita Classic^®^ H and Vita 3D Master^®^ Guides, as well as specific shades for teeth treated with bleaching [17,18]).

In the latest updates on spectrophotometers, we find Rayplicker Cobra^®^ (Borea, Limoges, France). This is a wireless spectrophotometer that measures and analyses wavelengths, comparing them with a database to determine the tooth’s colour. The measurement can be performed on any part of the tooth. It performs an analysis between 380 nm and 780 nm. To define the colour, it uses the CIELAB, which expresses the colour based on the three values of the tooth, allowing for colour deviations and precise luminosity studies. It allows for three-dimensional analysis similar to the functioning of the human eye and direct transmission of results to computer software [19,20].

Easyshade Advance 4.0 has been extensively validated in clinical repeatability studies. By contrast, clinical evidence on the Rayplicker Cobra remains comparatively limited: previous work has evaluated an earlier generation of the device, the Rayplicker Sandy, or has assessed repeatability across several intraoral spectrophotometers without a dedicated head-to-head comparison against Easyshade Advance 4.0 under a shared clinical protocol. This gap in the literature justifies the present clinical comparison between the two devices.

The primary aim of this study was to evaluate the intra-device repeatability of two spectrophotometric devices (Easyshade Advance 4.0 and Rayplicker Cobra) in dental shade determination. As secondary aims, the study assessed the agreement between the two devices and the agreement between each device and conventional visual shade selection using the Vita Classic Guide. For the primary outcome of intra-device strict repeatability, no inferential null hypothesis was specified because repeatability was analysed descriptively. For the secondary agreement analyses, the null hypotheses were that there was no agreement beyond chance (κ = 0) between (1) Easyshade Advance 4.0 and Rayplicker Cobra; (2) Easyshade Advance 4.0 and VITA Classical visual shade selection; and (3) Rayplicker Cobra and VITA Classical visual shade selection at each predefined tooth region. The corresponding alternative hypotheses were that agreement beyond chance was present (κ ≠ 0).

## 2. Materials and Methods

### 2.1. Study Design, Setting and Participants

A descriptive, cross-sectional observational study was conducted at the Alfonso X El Sabio University Clinic in Madrid, Spain. The study was approved by the UAX Ethics and Research Committee (2023_12/233; 15 December 2023). Selected patients completed an informed consent document for participation in the study.

The following inclusion criteria were followed for the selection of participants: Patients over 18 years of age, who retain teeth 2.1, 2.3 and 2.6, with periodontally healthy teeth, without prosthetic restorations, without mineralization disorders, without endodontic treatment and with good oral hygiene.

No formal a priori sample size calculation was performed. A sample of 35 patients was chosen based on the sample sizes used in the studies published by Czigola et al. and Abu-Hossin et al. [17,21]. In each patient, 3 teeth were analysed, resulting in a total of 105 teeth studied. Because three teeth were obtained from each patient, these 105 measurements are not fully independent observations; the patient was considered the statistical unit of clustering, and this within-patient correlation is acknowledged as a limitation of the statistical analysis (see Section 4).

### 2.2. Tooth Sites and Clinical Standardization

**Figure 1:**
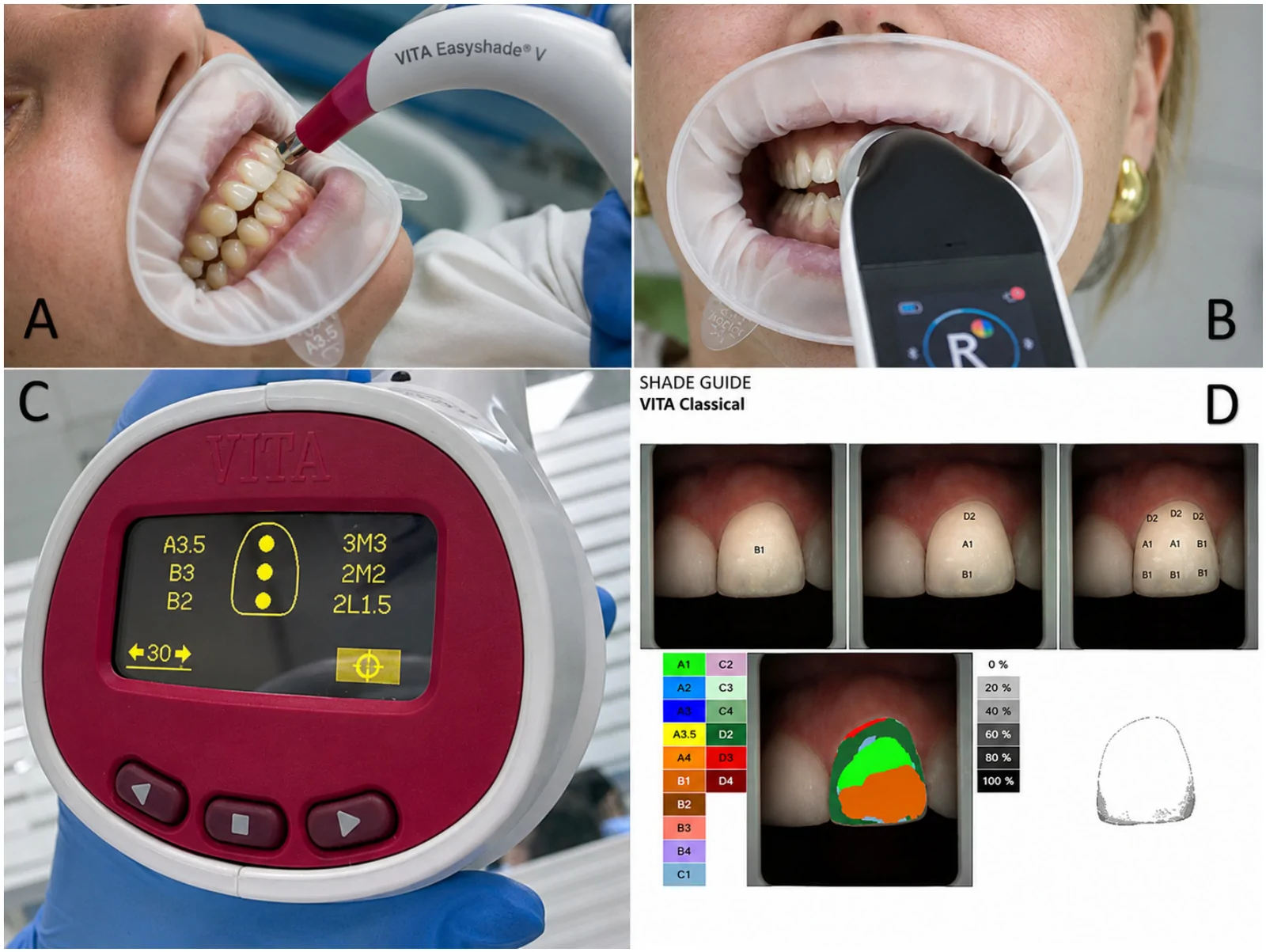
(**A**) Color capture with EasyShade 4.0. (**B**) Color capture with Rayplicker Cobra. (**C**) Results of Easyshade 4.0. (**D**) Results of Rayplicker Cobra.

Teeth 2.1, 2.3, and 2.6 were analysed, having been previously cleaned with a prophylaxis brush without any polishing paste. For the measurement, the anatomical center of the crown was determined in the cervical, middle, and incisal thirds of teeth 2.1 and 2.3, taking 3 shade measurements using the different measurement methods (1. Vita Classic Guide, 2. Vita Easyshade Advance 4.0, 3. Rayplicker Cobra) on the vestibular surfaces (Figure 1). In tooth 2.6, the shade was only taken in the middle third of the vestibular surface, as this tooth does not pose as much of an aesthetic challenge as the anterior sector.

The participants remained in the same clinical position throughout the sequence, and the tooth surfaces were kept hydrated.

### 2.3. Visual Shade Selection and Instrumental Shade Determination

Each instrumental shade measurement (Easyshade (VITA Zahnfabrik, Bad Säckingen, Germany) and Rayplicker (Borea, Limoges, France)) was repeated 3 times to verify the reliability of the test; the visual assessment with the Vita Classic Guide was performed only once per site, so intra-examiner repeatability of the visual method could not be calculated, which is acknowledged as a limitation. Additional data were collected from each patient, including age, regular consumption of coffee, wine, and/or tea, smoker status, prior whitening, and medication use and which ones.

All measurements, both visual and instrumental, were performed by a single operator (L.P.-R.), a dentist who had been specifically instructed in the use of both spectrophotometric devices prior to data collection, to minimize intra-operator variability, following a design comparable to previous clinical repeatability studies [22,23]. For each spectrophotometric reading, the probe tip was positioned perpendicular to the vestibular surface with stable, consistent contact at the selected point, and the device was completely removed from the mouth and repositioned between each of the three repeated measurements [24]; the interval between repetitions was not fixed in advance but corresponded to the time needed to reposition and reactivate the device, typically a few seconds, which is acknowledged as a source of minor, non-standardized timing variability. Both devices were calibrated according to the manufacturer’s instructions immediately before each measurement session [25]. All measurements were performed under standardized operatory illumination of approximately 5500 K against a neutral background [6,23], and were completed within the first minute after prophylaxis to minimize the confounding effect of enamel dehydration, which can produce a measurable increase in tooth value within seconds of isolation [26]. Visual shade selection with the Vita Classic Guide was always performed first, before the Easyshade and Rayplicker readings were obtained, so that the operator remained blinded to the instrumental results at the time of the visual assessment. The same single operator, whose normal colour vision had been confirmed with the Ishihara test, performed all visual evaluations [27]. The shade guide was arranged in ascending order of value rather than in its default alphanumeric order, consistent with recommended shade-selection protocols [28], and the shade tabs were disinfected between patients. Instrumental shade-determination technology continues to diversify, from professional spectrophotometers to smartphone-based applications [29], underscoring the need for standardized acquisition protocols such as the one followed here. Throughout the procedure, patients remained seated in an upright position in the dental chair, with the head stabilized against the headrest, consistent with recommended positioning for clinical shade-matching studies [30].

Data analysis was carried out focusing on three basic lines:

1. Analysis of the accuracy of the three colour shots taken on each of the machines for each user.
2. Matching of the two machines analyzed in each shot.
3. Consistency of the colour shots from each machine with the reference guide.

### 2.4. Outcome and Statistical Analysis

To analyze the strict repeatability of each spectrophotometer, a dichotomous variable was created, classifying each participant as concordant if the shade designation matched across all three repeated shots at a given site, and as discordant otherwise. This variable was analyzed descriptively, obtaining the total number of concordant cases (strict repeatability) and the corresponding percentage in relation to the 35 participants. This measure reflects complete agreement across three repeated measurements taken under essentially the same conditions (i.e., repeatability) and should not be interpreted as a measure of accuracy relative to an external reference standard.

The analysis of agreement between machines was performed, on the one hand, through a descriptive analysis of the level of similarity for each of the shots in each of the areas and parts within the shots taken. In addition, statistical analysis was performed using Cohen’s Kappa index of agreement, which allows determining the degree of agreement between two variables. Values above 0.8 indicate excellent agreement, between 0.6 and 0.8 indicate good agreement, and less than 0.4 indicate poor agreement.

To study the degree of agreement between each spectrophotometer and the Vita Classic Guide, the Kappa index of agreement was used, in addition to descriptive analysis of the percentage of agreement.

After data collection was entered into the Excel program, statistical analysis was performed using SPSS (version 29.01.0).

## 3. Results

### 3.1. Analysis of the Strict Repeatability of Spectrophotometers

The overall strict repeatability rate (i.e., the percentage of participants for whom all three repeated measurements yielded the same shade designation) for the Easyshade Advance 4.0 device was 52.2%, with the highest strict repeatability occurring on tooth 2.1 in the middle zone and on the same tooth in the incisal zone. The overall strict repeatability rate for the Rayplicker Cobra device was 53%. The highest strict repeatability occurred on teeth 21 and 23, concentrated in the incisal third. However, the tooth with the highest percentage of reproducibility was in the middle zone of 2.1 (Table 1).

### 3.2. Agreement Between Easyshade 4.0 and Rayplicker Cobra (Inter-Device Agreement)

The results obtained from the analysis of agreement and equality between the Easyshade and Cobra spectrophotometers are shown in Table 2. Descriptively, the highest percentages of accuracy were found in tooth 2.1 in the middle and incisal areas. Statistically, all Kappa coefficients were significant (*p* < 0.05), and the null hypothesis of agreement could be rejected in every analysis; however, the magnitude of the Kappa coefficients was low to moderate throughout (0.053–0.386), so statistical significance should not be interpreted as evidence of strong inter-device agreement.

### 3.3. Agreement Between Each Spectrophotometer and Visual Shade Selection (Vita Classic Guide)

Table 3 shows the results of concordance and equality between the Vita Classic Guide and the Easyshade 4.0 spectrophotometer. At a descriptive level, the highest percentages were observed in the following cases: middle zone, tooth 2.1; incisal zone, tooth 2.1; middle zone, tooth 2.3; middle zone, tooth 2.6. However, no agreement was observed in the cervical zone of tooth 2.3. Regarding Cohen’s Kappa analysis, significant differences were observed with *p* < 0.001 in all cases except in the concordance analysis for the cervical zone of tooth 2.3, rejecting the hypothesis of null agreement between the colour taken by Easyshade 4.0 and the Vita Guide.

Applying the thresholds predefined in Methods, good agreement (0.6–0.8) was found only in the middle zone of tooth 2.1 (k = 0.619). The incisal zone of tooth 2.1 (k = 0.475) and the middle zone of tooth 2.3 (k = 0.418) correspond to moderate agreement, below the 0.6 threshold for “good”. Agreement in the remaining cases is low, with Kappa levels below 0.4.

The analysis of the agreement between the Rayplicker Cobra and the Vita Guide is shown in Table 3. Only one case was recorded where the percentage of agreement was above 50%, and that was in the middle zone of piece 2.1. The null hypothesis of zero agreement was rejected in all cases; however, medium or high agreement was achieved in none of them. The highest agreement rates were found in the middle zone, piece 2.1 (k = 0.362), and the middle zone, piece 2.3 (k = 0.216).

## 4. Discussion

This exploratory clinical study evaluated the strict intra-device repeatability of Easyshade Advance 4.0 and Rayplicker Cobra after complete removal and repositioning between repeated readings and assessed agreement between the two devices with repeated visual VITA Classical shade selection. Both devices produced almost identical overall strict repeatability rates (52.2% for Easyshade and 53.0% for Rayplicker Cobra), but repeatability varied considerably according to tooth and measurement region. Inter-device agreement was predominantly poor (Kappa = 0.053–0.386), indicating that the two instruments cannot be regarded as interchangeable. Easyshade showed greater agreement with the Vita Classic guide in selected regions, particularly in the middle third of tooth 2.1 (Kappa = 0.619), whereas Rayplicker Cobra did not reach moderate agreement with the visual method in any region. These findings describe measurement stability and inter-method comparability; they do not establish absolute accuracy or trueness.

From a clinical perspective, tooth-shade determination is an important diagnostic step in restorative and prosthodontic treatment planning. The selected shade may influence material choice, ceramic layering, characterization, communication with the dental laboratory, and the final assessment of whether a restoration integrates with the adjacent dentition. The moderate repeatability observed in the present study means that a single instrumental reading may not adequately represent the colour of a tooth. Inconsistent measurements may contribute to an incorrect shade prescription, additional laboratory corrections, restoration remakes, increased treatment time, and patient dissatisfaction. Therefore, the clinical value of a shade-measuring device depends not only on its ability to generate an objective numerical or categorical result, but also on the stability of that result when the measurement is repeated under the same clinical conditions.

Electronic devices applied to colour measurement in dentistry play an important role that continues to be updated, with the presumed intention of being able to replace existing conventional methods, being able to achieve greater precision and facilitate the workflow [17,18].

The regional differences identified in this study are also clinically relevant. Repeatability varied markedly by anatomical region. Both devices performed best in the middle third of tooth 2.1, while lower values occurred in several cervical, incisal, and posterior sites. This pattern may be related to differences in tooth morphology and optical composition. The middle third usually provides a broader and more regular surface and is strongly influenced by the underlying dentine, while the cervical region may be affected by greater surface curvature, gingival proximity, and reduced accessibility. The incisal third is characterized by increased enamel translucency and a stronger influence of the oral background, whereas the convex vestibular surface of posterior teeth may make stable positioning of a contact probe more difficult. Consequently, device angulation, probe-tooth contact, and the exact measurement location may have a greater effect in these areas. Similar concerns regarding measurement geometry, surface characteristics, and clinical standardization have been discussed in previous studies and reviews [31,32,33,34,35]. Dehydration can also alter tooth appearance during clinical assessment [19]. In the present study, tooth surfaces were maintained hydrated, and the patient position was kept constant, but objective illuminance and colour temperature were not measured, and contact pressure/angulation were not instrumentally standardized.

Klotz AL et al. [33] studied the repeatability of results obtained with Easyshade 4 and Easyshade V, establishing that both devices are reliable and can be recommended in clinical practice. These results are broadly consistent with the repeatability observed for Easyshade Advance 4.0 in our study; however, given that overall strict repeatability in our sample was only 52.2%, our findings support a more cautious interpretation, in which Easyshade should be regarded as a useful diagnostic aid rather than as a device with demonstrated high reliability. Hampé-Kautz V et al. [34] established that the Rayplicker Sandy can be considered a competitor to Easyshade for optimal performance. The greater agreement between Easyshade and the Vita Classic guide in several regions should not be interpreted automatically as superior trueness. Visual assessment and instrumental measurement are based on different processes: the observer integrates the appearance of the entire tooth under a particular illumination, while the spectrophotometer samples a defined area and converts the reflected light into a shade designation according to its internal algorithm and shade library. Agreement with the visual method therefore indicates clinical comparability with current practice but does not establish that one method has identified the objectively correct shade. The existing literature reviewed has its own limitations [35,36]; the studies conducted use different population sample sizes and different shot repetitions for shade determination, making it difficult to clearly establish which shade-determination method is most effective, and further studies in this area are needed. Furthermore, there are numerous devices, and each study selects specific ones, so an overall comparison would be unclear. The authors agree that spectrophotometers are a reliable method for colour acquisition [37]. Previous investigations have likewise reported variable agreement between visual and digital procedures and have emphasized that neither approach is free from limitations [17,21,31,38]. In routine practice, the most defensible strategy is therefore to use instrumental measurements as an adjunct to, rather than an uncritical replacement for, a carefully standardized visual evaluation.

The findings support the use of a structured shade-selection protocol. Before measurement, the tooth should be cleaned without dehydrating the surface, the instrument should be calibrated according to the manufacturer’s instructions, and measurements should be obtained promptly under controlled illumination and against a neutral background. The probe should be positioned perpendicular to the selected region with stable contact, and at least two or three readings should be recorded. If the readings disagree, the measurement should be repeated and checked visually by a trained observer. Dehydration, environmental illumination, background colour, and observer-related factors can alter shade assessment [6,10,11,15,19,35]. For laboratory communication, the categorical shade should ideally be accompanied by the measured tooth region, standardized clinical photographs, and, when available, numerical colour information. Importantly, results generated by different devices should not be substituted for one another without prior validation, because the low inter-device Kappa values observed here demonstrate clinically meaningful device-dependent variation. Therefore, a laboratory prescription or longitudinal clinical follow-up should ideally use the same validated device rather than assuming that a shade designation from one system is equivalent to the designation produced by another.

The present results are generally consistent with the literature showing that spectrophotometers can improve standardization but do not eliminate variability. Kalantari et al. reported comparable performance for two spectrophotometric systems relative to a visual guide [31], while Klotz et al. found clinically acceptable reliability for Easyshade models under laboratory and clinical conditions [33]. More recently, Hampé-Kautz et al. demonstrated that repeatability differs among intraoral spectrophotometers and according to the shade system used [32]. Differences between published studies may reflect the device generation, calibration procedure, tooth type, shade guide, number of repetitions, operator experience, hydration state, and statistical definition of repeatability. Rayplicker Cobra is a more recently introduced device than the systems evaluated in most previous publications; consequently, the present clinical comparison provides useful preliminary evidence, but direct comparison with earlier Rayplicker models should be made cautiously [39,40]. As outlined in the Introduction, direct clinical evidence comparing Rayplicker Cobra with a well-established device such as Easyshade Advance 4.0, under a shared protocol and regional stratification by tooth third, was previously lacking; the present study addresses this specific gap rather than repeating comparisons already available for other device pairings.

Several limitations must be considered. No formal a priori sample size or power calculation was performed for the Kappa-based agreement analyses; the sample of 35 patients was based on the sample sizes used in previous comparable studies, and the adequacy of this sample size for detecting the observed Kappa levels cannot be assumed. In addition, three teeth were analysed per patient, so the 105 measurements are not fully independent observations; the statistical analyses were not adjusted for this within-patient clustering, which should be taken into account when interpreting the precision of the reported percentages and Kappa coefficients. Age, tobacco use, coffee/wine/tea consumption, prior bleaching, and medication use were recorded for each participant for descriptive purposes (see Section 2), but were not analysed, reported in the Results, or entered as covariates in the statistical analysis; patient sex was not collected as a study variable. Given the recognized influence of some of these factors on the optical properties of enamel and dentine, future studies should formally analyze them as potential covariates. The study was conducted in a single clinical centre with 35 adults and evaluated only three maxillary tooth types, which limits generalizability to other populations, teeth, and clinical situations. Shade results were analysed as Vita Classic categories, and agreement was assessed with Cohen’s Kappa; continuous CIELAB coordinates and perceptual colour differences calculated with CIEDE2000 were not available. Consequently, two measurements classified into different shade tabs may have been visually close, whereas identical categorical classifications may still conceal relevant numerical differences. In addition, the Vita Classic visual assessment was not an independent instrumental gold standard, so the study cannot establish the absolute trueness of either spectrophotometer. The strict definition of repeatability required all three readings to be identical and may therefore have produced lower percentages than methods based on numerical colour tolerances. Operator-related variability, the effect of repeated repositioning, and the impact of the measurements on the shade match of the final restoration were not evaluated. Furthermore, confidence intervals for the repeatability percentages and Cohen’s Kappa coefficients were not included in the original statistical analysis. Consequently, the precision of these estimates cannot be formally quantified, and future prospective studies should incorporate confidence intervals together with agreement coefficients to improve the interpretation of the results. In addition, the visual assessment with the Vita Classic Guide was performed only once per site; therefore, intra-examiner reliability of the visual method could not be evaluated. Consequently, the repeatability of the visual comparator itself, and its potential contribution to the device-versus-guide agreement reported in Table 3, remain unknown. Finally, this study cannot determine which of the two spectrophotometers, or the visual method, provides measurements closer to the true tooth colour. Addressing this question would require an independent instrumental reference standard, such as a calibrated spectroradiometer or certified physical colour standards, which was beyond the scope of the present clinical comparison [41].

Despite these limitations, this study shows that Easyshade Advance 4.0 and Rayplicker Cobra, although both purpose-built spectrophotometers, are not interchangeable with one another, given the predominantly poor inter-device Kappa values observed. The visual assessment with the Vita Classic Guide was performed only once per site, so intra-examiner reliability for the visual method could not be calculated; consequently, the repeatability of the visual comparator itself, and its potential contribution to the device-vs-guide agreement reported in Table 3, could not be evaluated. Consequently, this study cannot determine which of the two spectrophotometers, or the visual method, provides measurements closer to the true tooth colour; addressing this question would require an independent instrumental reference standard, such as a calibrated spectroradiometer or certified physical colour standards, which was beyond the scope of the present clinical comparison [41].

Although several participant-related variables (e.g., previous tooth whitening, smoking status, consumption of chromogenic beverages, and medication use) were recorded during data collection, they were not included as explanatory variables in the statistical analysis. Future studies should evaluate their potential influence on shade determination using multivariable analytical approaches.

No intraoral scanner was evaluated in the present study, and therefore no comparison with scanner-based shade determination can be drawn from these data; further clinical studies, including scanner-based systems and an independent reference standard, are warranted to clarify this question.

Future studies should include larger and multicenter samples, multiple trained operators, standardized illumination and hydration protocols, and a wider range of anterior and posterior teeth. Comparisons should incorporate an independently calibrated reference instrument, continuous CIELAB measurements, CIEDE2000 colour differences, and predefined perceptibility and clinical acceptability thresholds. It would also be valuable to determine whether the use of repeated instrumental measurements, combined visual–digital protocols, or device-specific laboratory communication reduces restoration remakes and improves clinician- and patient-reported aesthetic outcomes. Such studies would clarify whether newer spectrophotometers provide a clinically meaningful diagnostic advantage rather than only a different method of assigning a shade category.

## 5. Conclusions

Under the conditions of this clinical study, Easyshade Advance 4.0 and Rayplicker Cobra showed similar descriptive strict repeatability percentages, with identical categorical shade results in all three repeated measurements at approximately half of the evaluated participant-site combinations. This descriptive similarity should not be interpreted as statistical equivalence. Agreement between the two devices was predominantly slight to fair, indicating that their categorical shade designations should not be considered interchangeable.

Easyshade showed greater agreement than Rayplicker Cobra with the consensus VITA Classical visual method in selected tooth regions, particularly the middle third of tooth 2.1. Because the visual method was a subjective clinical comparator and no independent instrumental reference standard was used, these findings do not establish superior absolute trueness. Both devices are best interpreted as adjuncts within a standardized, repeated shade-selection workflow, and further well-powered studies using controlled illumination, raw colorimetric outcomes, and appropriate clustered statistical methods are required.

## Figures and Tables

**Table 1:** Analysis of the strict repeatability of the Easyshade 4.0 and Rayplicker Cobra devices.

	Cervical		Medium			Incisal	
Device	2.1	2.3	2.1	2.3	2.6	2.1	2.3
**Easyshade 4.0**	11	14	27	19	15	25	17
**Strict repeatability %**	31.4%	40.0%	77.1%	54.3%	42.9%	71.4%	48.6%
**Rayplicker Cobra**	14	17	22	19	16	21	21
**Strict repeatability %**	40.0%	48.6%	62.9%	54.3%	45.7%	60.0%	60.0%

**Table 2:** Analysis of concordance between Easyshade 4.0 and Cobra.

		Cervical		Medium			Incisal	
		2.1	2.3	2.1	2.3	2.6	2.1	2.3
**% Match**		33.3%	19.0%	57.1%	28.6%	7.6%	45.7%	34.3%
**Kappa**	Valor	0.203	0.079	0.386	0.202	0.053	0.161	0.241
	*p*	<0.001	<0.05	<0.001	<0.001	<0.001	<0.001	<0.001

**Table 3:** Analysis of concordance between each spectrophotometer (Easyshade 4.0, Rayplicker Cobra) and the Vita Classic Guide.

	Cervical		Medium			Incisal	
Device/Metric	2.1	2.3	2.1	2.3	2.6	2.1	2.3
**Easyshade 4.0 vs. Vita Classic**							
**% Match**	30.5%	10.5%	74.3%	49.5%	48.6%	64.8%	31.4%
**Kappa (*p*)**	0.136 (0.001)	0.010 (0.721)	0.619 (<0.001)	0.418 (<0.001)	0.360 (<0.001)	0.475 (<0.001)	0.188 (<0.001)
**Rayplicker Cobra vs. Vita Classic**							
**% Match**	27.6%	23.8%	54.3%	24.8%	6.7%	41.0%	24.8%
**Kappa (*p*)**	0.149 (<0.001)	0.118 (<0.001)	0.362 (<0.001)	0.216 (<0.001)	0.038 (0.010)	0.129 (0.003)	0.158 (<0.001)

## Data Availability

The original contributions presented in the study are included in the article. Further inquiries can be directed to the corresponding author.
